# TRIB2 functions as novel oncogene in colorectal cancer by blocking cellular senescence through AP4/p21 signaling

**DOI:** 10.1186/s12943-018-0922-x

**Published:** 2018-12-12

**Authors:** Zhenlin Hou, Kaixuan Guo, Xuling Sun, Fuqing Hu, Qianzhi Chen, Xuelai Luo, Guihua Wang, Junbo Hu, Li Sun

**Affiliations:** 1 Cancer Research Institute, Tongji Hospital, Huazhong University of Science and Technology, Wuhan, China; 2 Department of Oncology, Tongji Hospital, Huazhong University of Science and Technology, 1095 Jiefang Av, Wuhan, Hubei 430030 People’s Republic of China

**Keywords:** Colorectal cancer, Cellular senescence, TRIB2, AP4, p53, p21

## Abstract

**Background:**

Cellular senescence is a state of irreversible cell growth arrest and senescence cells permanently lose proliferation potential. Induction of cellular senescence might be a novel therapy for cancer cells. TRIB2 has been reported to participate in regulating proliferation and drug resistance of various cancer cells. However, the role of TRIB2 in cellular senescence of colorectal cancer (CRC) and its molecular mechanism remains unclear.

**Methods:**

The expression of TRIB2 in colorectal cancer tissues and adjacent tissues was detected by immunohistochemistry and RT-PCR. The growth, cell cycle distribution and cellular senescence of colorectal cancer cells were evaluated by Cell Counting Kit-8 (CCK8) assay, flow cytometry detection and senescence-associated β-galactosidase staining, respectively. Western blot, RT-PCR and luciferase assay were performed to determine how TRIB2 regulates p21. Immunoprecipitation (IP) and chromatin-immunoprecipitation (ChIP) were used to investigate the molecular mechanisms.

**Results:**

We found that TRIB2 expression was elevated in CRC tissues compared to normal adjacent tissues and high TRIB2 expression indicated poor prognosis of CRC patients. Functionally, depletion of TRIB2 inhibited cancer cells proliferation, induced cell cycle arrest and promoted cellular senescence, whereas overexpression of TRIB2 accelerated cell growth, cell cycle progression and blocked cellular senescence. Further studies showed that TRIB2 physically interacted with AP4 and inhibited p21 expression through enhancing transcription activities of AP4. The rescue experiments indicated that silencing of AP4 abrogated the inhibition of cellular senescence induced by TRIB2 overexpression.

**Conclusion:**

These data demonstrate that TRIB2 suppresses cellular senescence through interaction with AP4 to down-regulate p21 expression. Therefore, TRIB2 could be a potential target for CRC treatment.

**Electronic supplementary material:**

The online version of this article (10.1186/s12943-018-0922-x) contains supplementary material, which is available to authorized users.

## Background

Tribbles homolog 2, a member of the tribbles family (TRIB1, TRIB2, TRIB3), is first identified in *Drosophila* as mitosis blocker that regulates embryo and germ cell development [1]. It comprises an N-terminal domain, a C-terminal domain, and a central pseudokinase domain that contains a Ser/Thr protein kinase-like domain but lacks ATP affinity and catalytic activity [2]. In the absence of kinase activity, TRIB2 functions as a scaffold protein to regulate different signaling pathway in fundamental biological processes as well as in pathological conditions, including cancer [3]. TRIB2 plays a crucial role in regulating various cellular processes in cancer, such as proliferation, apoptosis and drug resistance [4–6]. Currently, the role of TRIB2 in cancer remains controversial. TRIB2 is overexpressed in human acute myeloid leukemia (AML) and accelerates AML progression via the inactivity of C/EBPα [7]. In liver cancer, TRIB2 functions as an adaptor protein and promotes YAP protein stabilization through the E3 ubiquitin ligase βTrCP, contributing to cancer cell proliferation and transformation [8]. In contrast, Mara et al. reported that TRIB2 might counteract the chemotherapy resistance and propagation in myeloid leukemia via activation of p38; in liver cancer, TRIB2 inhibits Wnt-signaling by regulating the degradation of key factors, such as βTrCP, COP1 and Smurf1 [6, 9]. Interestingly, recent literature has reported that high-TRIB2 expression correlated with a worse clinical outcome of colorectal cancer (CRC) [10]. However, the biological role of TRIB2 and its underlying mechanism in CRC are not fully understood.

Cellular senescence is a state of growth arrest and characterized as some phenotypic alterations, such as remodeled chromatin, reprogrammed metabolism, morphology changes and up-regulated senescence-associated β-galactosidase (SA-β-gal) activity [11, 12]. Various intrinsic and extrinsic insults could trigger cellular senescence, including oxidative stress, mitochondrial dysfunction, DNA damage and therapeutic drugs or radiation [13]. Substantial evidence has shown that disruption of senescence accelerates and induction of senescence inhibits cancer development [14]. Therefore, senescence might be a promising target for tumor therapy.

The cyclin-dependent kinase inhibitor p21 (CDKN1A or p21^WAF1/Cip1^), a member of the Cip/Kip family, is a critical regulator of cell cycle exit and cellular senescence through blocking the activities of cyclin-dependent kinases (CDK), including CDK1 and CDK2 [15–17]. Microarray-based studies indicate that p21 is positively correlated with genes involved in cellular senescence [18]. Currently, induction of p21 expression by a variety of stimuli is thought to be the driver of senescence initiation [19]. The tumor suppressor protein p53 is the major transcription regulator for p21 and multiple proteins involved in regulating cellular senescence work through p53/p21 pathway. Besides, many other transcription factors like Smad3, BRCA1, CHK2 and transcription factor activating enhancer-binding protein 4 (AP4), have been reported to control p21 expression [20, 21]. As a member of the basic helix-loop-helix transcription factors superfamily, AP4 activates or represses a series of genes by recognizing and binding to the E-box sequence CAGCTG in the promoter [22]. It has been reported that AP4 occupies the four CAGCTG motifs in the promoter of p21 and subsequently repressing its transcription activity to contribute to cancer cell proliferation and cell cycle arrest [21, 23].

In the present study, we found that TRIB2 was overexpressed in colorectal cancer and inversely correlated with survival rate of CRC patients. Down-regulation of TRIB2 inhibited cancer cells proliferation, induced cell cycle arrest and promoted senescence in CRC cells. Moreover, TRIB2 physically interacted with AP4 and the TRIB2-AP4 interaction enhanced AP4-mediated transcriptional activity. Using rescue experiments, we demonstrated TRIB2 negatively regulated cellular senescence through cooperating with AP4 to repress p21 expression. Thus, our study identifies a novel mechanism mediated by TRIB2/AP4/P21 axis in regulating cellular senescence, and suggests that TRIB2 might be a new target in clinical practice for CRC treatment.

## Materials and methods

### Colorectal cancer samples

Primary tumor samples and the corresponding adjacent normal tissues were obtained from CRC patients who received surgical resection at Tongji Hospital (Wuhan, China), between January 2017 and January 2018, after their written informed consent. None of the patients received chemotherapy or radiotherapy before surgery. This study was approved by the Huazhong University of Science and Technology Ethics Committee.

### Cell lines, antibodies and reagents

The cell lines HEK 293 T, SW48 and LoVo were obtained from the American Type Culture Collection (ATCC, Manassas, VA, USA). These cells were cultured in Dulbecco’s modified Eagle’s medium (DMEM) plus 10% fetal bovine serum (FBS) at 5% CO2 and 37 °C. The antibodies against p53 (sc-47698) and p21 (sc-397) were purchased from Santa Cruz Company (Santa Cruz, CA, USA), the antibody against GAPDH (A00227) were purchased from Boster Company (Boster, Wuhan, China), the antibody to TRIB2 (A11661) were purchased from Abclonal Company (Abclonal, Wuhan, China) and the antibody to AP4 (ab28512) were purchased from Abcam Company (Abcam, MA, USA). Doxorubicin (Dox), used to establish the cellular senescent model, was obtained from Calbiochem (La Jolla, CA, USA).

### Cell viability assay

Cell viability was determined by CCK8 assays. Briefly, colon cancer cells were seeded in 96-well plates (5 × 10^3^ cells/well) and treated with corresponding processes. CCK8 was added into the wells for 3 h at indicated times. The absorbance in each well at wavelength of 450 nm (A450) was measured with a Thermomax microplate reader.

### Cell cycle analysis

Cells were trypsinized, washed with cold phosphate-buffered saline (PBS) and fixed in 80% ethanol overnight at − 20 °C. Cells were then washed twice with PBS, and stained with PI at room temperature for 1 h. Cell cycle distribution was measured by the Becton-Dickinson FACScan System (Franklin Lakes, NJ, USA).

### Apoptosis assay

Cells were trypsinized, washed with cold phosphate-buffered saline (PBS), stained with Annexin V-FITC and propidiumiodide (PI) using an Annexin V-FITC/PI-staining kit (BD Pharmingen, San Diego, CA, USA) and placed at room temperature for 30 min. The apoptosis of cells was measured by flow cytometry.

### Senescence-associated β-galactosidase staining

Tumor cells were transfected with or without siRNA or plasmid, treated with doxorubicin and cultured for 48 h. Cells were washed with PBS for 3 times and stained with freshly prepared SA-β-Gal staining solution following the protocol provided by the manufacturer (Beyotime Biotechnology Ltd., Shanghai, China). The stained cells were detected with a microscope and the percentage of senescence cells was quantified by calculating the percentage of SA-β-Gal-positive cells in randomly selected fields (*n* = 3).

### Western blot analysis and Immunoprecipitation (IP)

Cancer cells were collected, washed twice with cold PBS and lysed in NP-40 lysis buffer for 30 min at 4 °C. Protein concentration was measured using bicinchoninic acid assay kit (Thermo). Protein extracts were separated by electrophoresis in an 8~12% premade sodium dodecyl sulfate-polyacrylamide minigel (Tris-HCL SDS-PAGE) and transferred to a PVDF membrane. The membrane was incubated with indicated antibodies and detected by using a chemiluminescence method. For immunoprecipitation, total cell lysates were incubated with appropriate antibodies overnight and subsequently rotated with protein A/G beads for 2~4 h at 4 °C. Beads were washed three times using NP-40 lysis buffer, mixed with 2 × SDS sample buffer and boiled for 5~10 min. The co-precipitates were analyzed by western blot analysis.

### Immunohistochemistry (IHC)

The procedures followed standard manufacturer’s protocols as described previously. Two pathologists reviewed and scored IHC staining for each sample independently. IRS system was used to quantify IHC staining. The percentage of positively stained tumor cells was scored as follows: 1 (< 10%), 2 (10–50%), 3 (50–75%) and 4 (> 75%). Staining intensity was scored 0–3: 0, no staining; 1, weak staining (light yellow); 2, moderate staining (yellow brown); 3, strong staining (brown). The staining index ranged from 0 to 12, which was calculated by multiplying the score of the percentage of positive tumor cells and the staining intensity.

### GST pull-down assay

GST-TRIB2 fusion proteins and His-AP4 proteins were expressed in BL21 and purified using Glutathione Sepharose 4B beads (Amersham Pharmacia, Piscataway, NJ, USA) or Ni beads (GE Healthcare, CA, USA), respectively. Purified His-AP4 protein was incubated with GST or GST-TRIB2 fusion proteins bound to Glutathione Sepharose beads at 4 °C overnight. Beads-associated proteins were detected by western blot. Expression of GST fusion proteins was confirmed by Coomassie Blue staining.

### Luciferase activity assay

Cancer cells were seeded in 12-well plates (2 × 10^5^ cells/well) and co-transfected with luciferase reporter constructs contained p21 promoter (p21-Luc), TK-Renilla expression plasmids, along with indicated expression plasmids or siRNA by lipofectamine 2000 reagent. After 48 h of transfection, luciferase activity assay was performed using the dual luciferase assay kit according to the manufacturer’s instruction (Promega, Madison, WI, USA). Firefly luciferase activity was normalized to Renilla luciferase activity. All the experiments were carried out three times.

### ChIP assay

ChIP assay was carried out using the ChIP assay kit according to the protocol. Briefly, 1 × 10^7^ cancer cells were harvested and treated with 1% formaldehyde for 10 min at 37 °C to cross-link. To stop the reaction, glycine was added to the cell suspension at a final concentration of 0.125 M. Chromatin was sheared on ice by sonication to generate DNA fragments with a bulk size of 200~1000 bp. After centrifugation, the cell lysates were incubated with indicated antibody overnight and subsequently with protein G-agarose beads for 2~4 h at 4 °C with agitation. Beads were washed and eluted, and the cross links were reversed by incubation at 65 °C for 4 h. Purified DNA was used to analyze the binding of Flag-AP4 or His-TRIB2 to p21 promoter locus by PCR reactions. The sequences of oligonucleotides used as qChIP primers: p21 promoter forward 5’-TGTGTCCTCCTGGAGAGTGC-3′ and p21 promoter reverse 5’-CAGTCCCTCGCCTGCGTTG-3′.

### RNA interference

Short interfering RNA (siRNA) oligonucleotide duplexes targeting TRIB2, AP4 and p53 used in this study were synthesized and purified by RiboBio (Ribobio Co., Guangzhou, China). The sequences of TRIB2 (#1 and #2), AP4 and p53 are as follows.

siTRIB2 #1: 5′- CGTGGACTCTAGTATGTAAAT -3′,

siTRIB2 #2: 5′- GCGTTTCTTGTATCGGGAAAT -3’.

siAP4#1: 5′- GTGATAGGAGGGCTCTGTAG -3’.

siAP4#2: 5’-GCAGAGCATCAACGCGGGATT -3′.

sip53: 5′- GACTCCAGTGGTAATCTAC -3′.

A nonsense siRNA with no homology to the known genes in human cells was used as negative control. siRNA transfections of cancer cells were performed by using Lipofectamine 2000 (Invitrogen, Carlsbad, CA, USA) according to the manufacturer’s instructions, and the knockdown efficiency was verified 48 h after transfection. All the siRNAs were used at a final concentration of 100 nM.

### Quantitative real-time PCR

Total RNA was extracted using the TRIzol (Invitrogen, Carlsbad, CA, USA) method in accordance with the manufacture’s protocol. Reverse transcription of total RNA was performed to generate cDNA. Real-time PCR was carried out using the Multi-color Real-Time PCR Detection System (Bio-Rad, Hercules, CA, USA) and SYBR Green Real-time PCR Master Mix (TOYOBO, Shanghai, China). The primer sequences for real-time PCR analysis are listed as follows.

**Table:** 

Gene	Forward primer (5′- to 3′)	Reverse primer (5′- to 3′)
TRIB2	ATGAACATACACAGGTCTACCCC	GGGCTGAAACTCTGGCTGG
p21	CGATGGAACTTCGACTTTGTCA	GCACAAGGGTACAAGACAGTG
GAPDH	GGAGCGAGATCCCTCCAAAAT	GGCTGTTGTCATACTTCTCATGG
p53	CAGCACATGACGGAGGTTGT	TCATCCAAATACTCCACACGC
AP4	GAGGGCTCTGTAGCCTTGC	GAATCCCGCGTTGATGCTCT

### Animal study

Cancer cells were collected, washed with PBS, resuspended in culture medium and mixed with Matrigel (BD Biosciences) at the ratio of 1:1. The nude mice were randomly divided into two groups and subcutaneously injected with the prepared cells above (1 × 10^5^ cells/mouse). After a week of injection, tumor size was measured with calipers every 4 days. At the end of the experiment, all mice were sacrificed and the tumors were isolated and weighed.

### Statistical analysis

All statistical analyses were performed using SPSS 24.0 (SPSS Inc.). All data were quantified as mean ± SD. Two-tailed Student’s t-test was used to evaluate the differences between two groups. Survival curves were plotted using Kaplan-Meier methodology with log-rank test on univariate analysis. On multivariate analysis, Cox proportional hazards model was used for analyzing prognostic factors. The cut-offs for the expression levels were identified by reference to the Human Protein Atlas (www.proteinatlas.org). Values of *p* < 0.05 were considered as statistically significant in all cases.

## Results

### TRIB2 is overexpressed in colorectal cancer

**Fig. 1:**
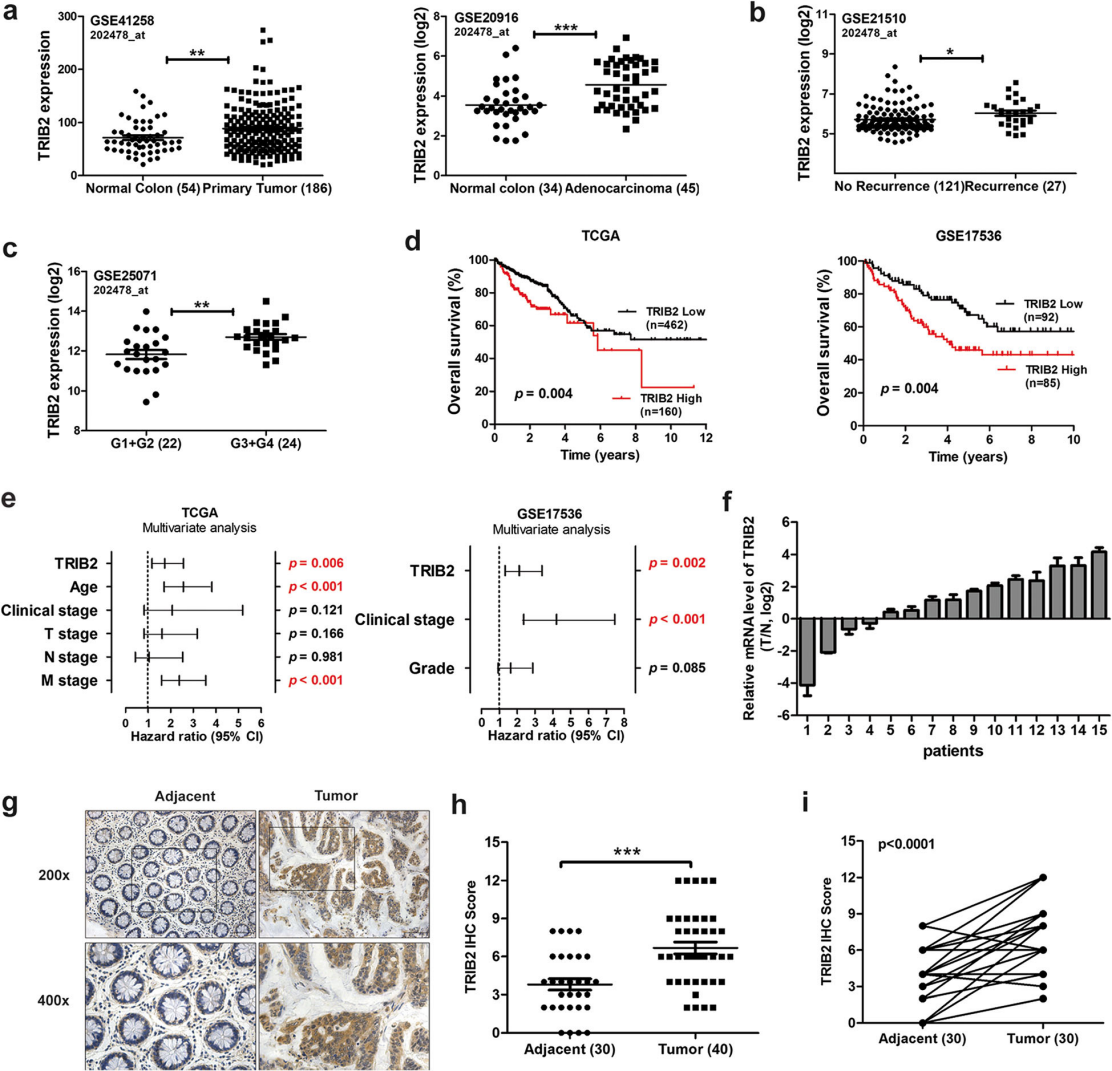
TRIB2 expression is elevated in CRC patients. **a** Analysis of TRIB2 expression in CRC and normal tissues from two colorectal cancer cohorts. The gene expression data (GSE41258, GSE20916) was downloaded from GEO database (** *p* < 0.01, *** *p* < 0.001, t-test). **b** Analysis of TRIB2 expression in CRC tissues from patients with or without recurrence. The gene expression data (GSE21510) was downloaded from GEO database (* *p* < 0.05, t-test). **c** Elevated TRIB2 expression was correlated with tumor grade. The gene expression data (GSE25071) was downloaded from GEO database (** *p* < 0.01, t-test). **d** Kaplan–Meier plot of overall Survival: patients with CRC (TCGA database and GSE17536 from GEO database) were stratified by TRIB2 expression level. **e** Cox regression analysis to evaluate the significance of the association between TRIB2 expression and CRC patients prognosis in the presence of other clinical variables. **f** mRNA expression of TRIB2 in 15 pairs of human primary CRC tissues (normal and Tumor). **g** Representative examples of TRIB2 staining in CRC and adjacent normal tissues. **h** and **i** Quantification of TRIB2 expression in normal and CRC samples

It has been reported that TRIB2 is amplified in human AML as well as other solid tumors [10, 24]. To determine the role of TRIB2 in CRC, we firstly analyzed the publically available human CRC datasets from Gene Expression Omnibus (GEO) databases, and found that TRIB2 was highly expressed in primary tumor tissues compared with adjacent normal tissues (Fig. 1a). CRC samples from patients with tumor recurrence expressed higher TRIB2 levels (Fig. 1b). We also found a significant association between TRIB2 expression and tumor grade (Fig. 1c). Further analysis of CRC datasets from both The Cancer Genome Atlas (TCGA) and GEO database showed that patients with high expression of TRIB2 suffered significantly worse overall survival (*p* = 0.004 and *p* = 0.004, respectively; Fig. 1d). Moreover, the multivariate analysis indicated that overexpression of TRIB2 was an independent prognosis factor for CRC patients (*p* = 0.006 and *p* = 0.002, respectively; Fig. 1e).

To corroborate these studies, we investigated TRIB2 mRNA expression in 15 pairs of CRC samples with normal colorectal tissues as control using RT-PCR. TRIB2 expression was higher in tumors than in adjacent tissues in 11 out of the 15 pairs (73.3%, Fig. 1f). IHC staining showed that the average expression level of TRIB2 was elevated in CRC tissues compared with normal tissues (*p* < 0.001, Fig. 1g and h). Further paired analysis also demonstrated that TRIB2 protein level was higher in tumors (76.7%, *p* < 0.001, Fig. 1i). Thus, these findings suggest that TRIB2 is highly expressed in a subset of CRC patients, and evaluated TRIB2 predicts poor clinical outcome.

### Knockdown of TRIB2 promotes cellular senescence and inhibits the growth of CRC cells

**Fig. 2:**
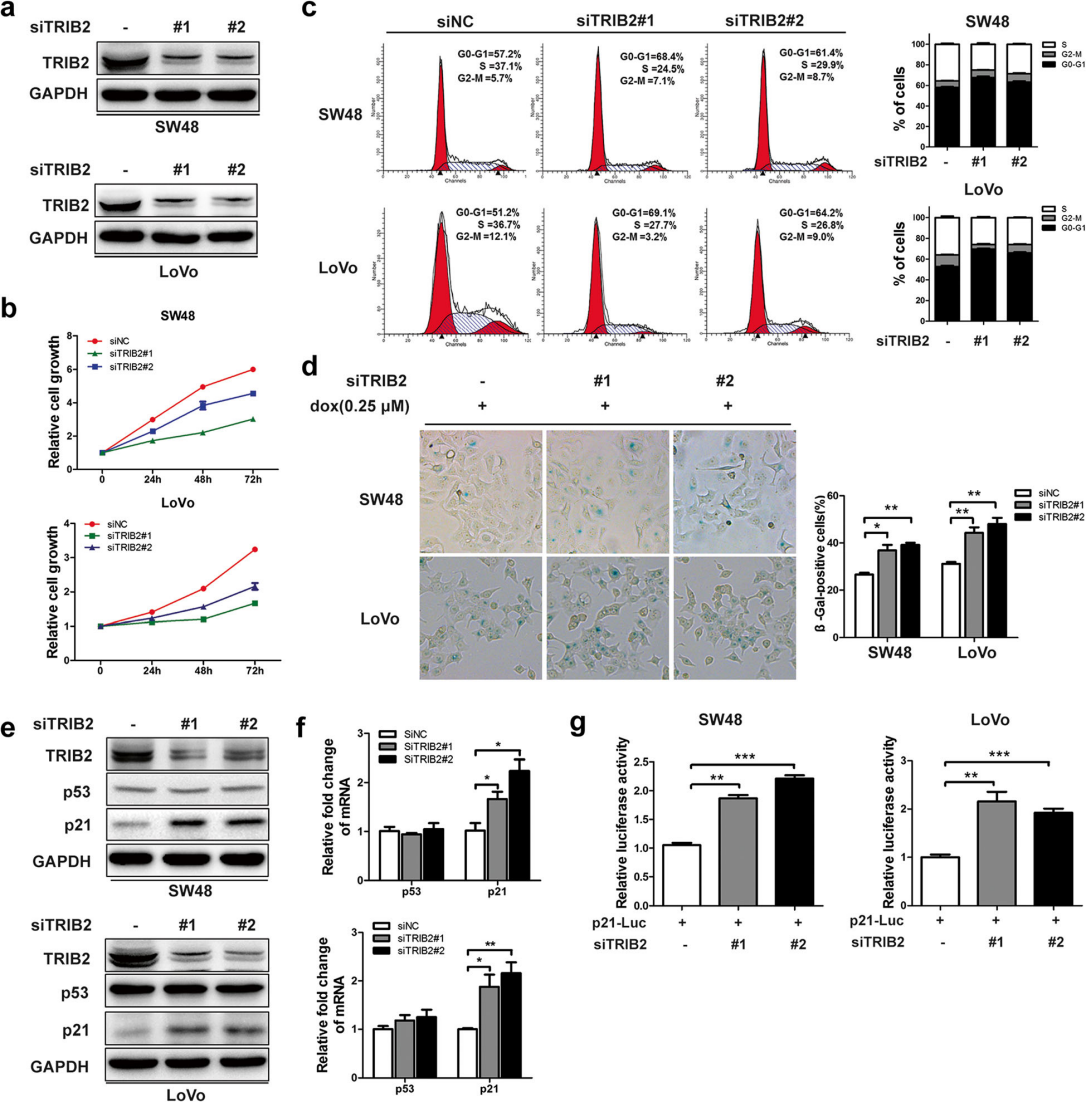
Knockdown of TRIB2 in CRC cells inhibits tumor cell growth and enhances cellular senescence. **a** Western blot of TRIB2 in SW48 and LoVo cells transfected with TRIB2-specific siRNA (siTRIB2#1 and siTRIB2#2) or control siRNA. **b** CCK8 analysis of cell viability in TRIB2-knockdown SW48 and LoVo cells at 0, 24, 48 and 72 h, respectively. **c** Cell cycle distribution measured by flow cytometry in TRIB2-knockdown SW48 and LoVo cells. **d** SA-β-gal staining analysis in TRIB2-knockdown SW48 and LoVo cells treated with dox (0.25 μmol/l, 48 h, left panel, representative images of SA-β-gal staining). **e** Western blot of TRIB2, p53 and p21 in SW48 and LoVo cells transfected with TRIB2-specific or control siRNA. **f** mRNA expression of p53 and p21 in SW48 and LoVo cells transfected with TRIB2-specific or control siRNA. **g** Luciferase activity of p21 promoter in SW48 and LoVo cells transiently transfected with p21-Luc plus TRIB2-specific or control siRNA. Results are presented as mean ± SD from three independent assays, * *p* < 0.05, ** *p* < 0.01, *** *p* < 0.001, t-test

And then, to test whether TRIB2 directly regulated the proliferation of CRC cells, we produced TRIB2 knockdown cell model by transfecting CRC cells with two siRNAs (Fig. 2a). CCK8 assay was performed to detect the effect of TRIB2 on cell proliferation. The results showed that depletion of TRIB2 significantly slowed down cell growth compared to control (Fig. 2b). To clarify whether defective cell cycle caused the inhibitory effect, cell cycle distribution of the cells transfected with TRIB2-specific siRNA were determined by flow cytometry. As shown in Fig. 2c, TRIB2 knockdown dramatically increased the G0/G1-phase ratios and reduced the S-phase ratios. Besides, we analyzed the effect of TRIB2 knockdown on cell apoptosis and found that silencing of TRIB2 could not induce apoptosis in SW48 and LoVo cells (Additional file 1: Figure S1a and b).

Since senescent cells have typical feature, such as low proliferation and cell cycle arrest, we asked whether the suppression of cell growth attributed to induction of senescence. We treated TRIB2-knockdown SW48 and LoVo cells with doxorubicin (dox, 0.25 μmol/l) for 48 h, and found that TRIB2 knockdown cells showed higher rates of flat and enlarged senescence-like morphology (data not shown). The level of SA-β-gal (a biomarker of senescence) was measured, and data showed that the percentage and strength of SA-β-gal-positive cells were significantly increased in TRIB2 knockdown condition (Fig. 2d). To explore the mechanism of suppression of cellular senescence mediated by TRIB2, we analyzed the expression of several senescence related genes, and found that TRIB2 knockdown significantly up-regulated the protein and mRNA expression of p21 (Fig. 2e and f). However, some other cell cycle regulatory molecules, such as cyclin D1 and p16, could not be regulated by silencing of TRIB2 (Additional file 2: Figure S2a). According to the results above, we speculated that TRIB2 regulated the expression of p21 at transcription level. We further performed luciferase assays using pGl3 containing p21 promoter (~ 2.0 kb) (p21-Luc). The luciferase activity of p21 promoter was stimulated by TRIB2 knockdown (Fig. 2g). On the contrary, overexpression of TRIB2 inhibited the mRNA and protein levels of p21, facilitated proliferation and cell cycle, and blocked cellular senescence in SW48 and LoVo cells. In addition, the luciferase activity of p21 promoter was markedly weakened in TRIB2 overexpressed SW48 and LoVo cells (Additional file 3: Figure S3). Our results show that TRIB2 is crucial for pro-proliferation and suppressing cellular senescence in CRC cells.

### TRIB2 regulates the expression of p21 in a p53-independent manner

**Fig. 3:**
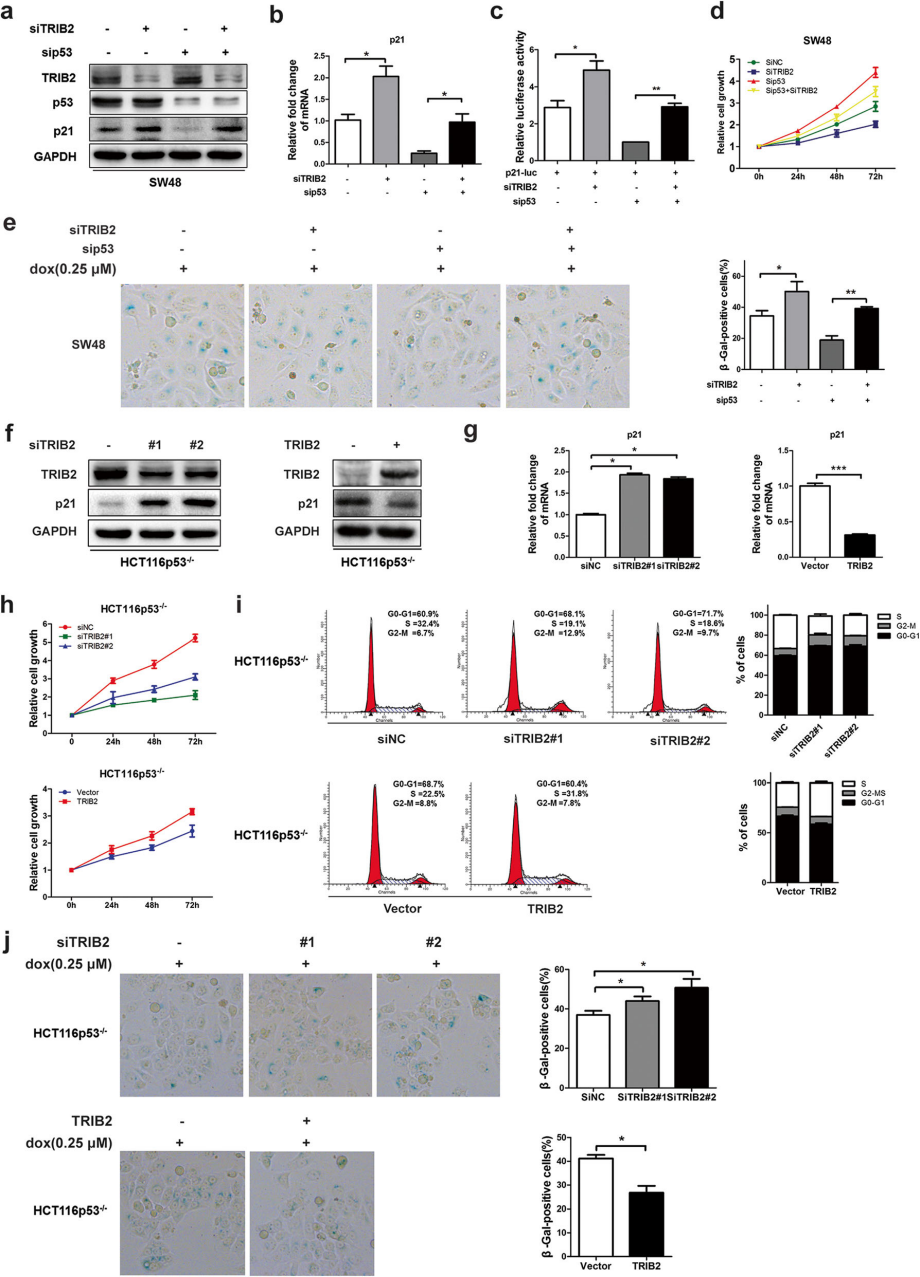
TRIB2 regulates p21 expression and cellular senescence in a p53-independent pathway. **a** Western blot of TRIB2, p53 and p21 in SW48 cells transfected with TRIB2- and or not p53-specific siRNA. **b** mRNA expression of p21 in SW48 cells transfected with TRIB2- and or not p53-specific siRNA. **c** Luciferase activity of p21 promoter in SW48 cells transfected with p21-Luc plus TRIB2- and or not p53-specific siRNA. **d** Silencing of TRIB2 inhibits cell proliferation in the absence of p53. SW48 cells were transfected with TRIB2- and or not p53-specific siRNA and the absorption (A450 nm) was detected at 0, 24, 48 and 72 h, respectively. **e** Silencing of TRIB2 promotes cellular senescence in the absence of p53. SW48 cells were transfected with TRIB2- and or not p53-specific siRNA and treated with dox (0.25 μmol/l) for 48 h. The percentage of SA-β-gal-positive cells was analyzed (right panel). **f** Western blot of TRIB2 and p21 in HCT116p53^−/−^ cells transfected with TRIB2-specific siRNA (#1 and #2) or TRIB2-expressing plasmid. **g** mRNA expression of p21 in HCT116p53^−/−^ cells transfected with TRIB2-specific siRNA (#1 and #2) or TRIB2-expressing plasmid. **h** Cell growth was analyzed by CCK8 in HCT116p53^−/−^ cells transfected with TRIB2-specific siRNA or TRIB2-expressing plasmid. **i** Cell cycle distribution was analyzed by flow cytometry in TRIB2-knockdown or -overexpressing HCT116p53^−/−^ cells. **j** SA-β-gal staining analysis in TRIB2-knockdown or –overexpressed HCT116p53^−/−^ cells treated with dox (0.25 μmol/l, 48 h). left panel, representative images of SA-β-gal staining; right panel, percentage of SA-β-gal-positive cells. Results are presented as mean ± SD from three independent assays, * *p* < 0.05, ** *p* < 0.01, *** *p* < 0.001, t-test

p53 is an important transcription factor involved in cell cycle inhibition and directly target the p21 promoter. We then examined levels of p53 in both TRIB2 knockdown/overexpressed and control cells, and found no significant changes in p53 expression at both protein and mRNA levels (Fig. 2e and f; Additional file 3: Figure S3e and f). To confirm TRIB2 regulated p21 expression in a p53-independent manner, we transfected p53-specific siRNA in TRIB2 knockdown SW48 cells to observe the p21 expression changes. As shown in Fig. 3 a and b, TRIB2 knockdown elevated the expression of p21 at protein and mRNA levels irrespective of p53 knockdown. The luciferase assays showed consistent results that the inhibitory effect of p21-luciferase activity by p53 knockdown was alleviated by silencing TRIB2 (Fig. 3 c). Moreover, silencing TRIB2 still inhibited cell growth and induced cellular senescence in p53 knockdown SW48 cells (Fig. 3 d and e). In parallel, we chose HCT116p53^−/−^ cells, a p53-null originating from HCT116 wt cells, and explored whether TRIB2 regulated p21 expression in these cells. As expected, no matter what the status of p53 was, silencing TRIB2 dramatically up-regulated p21 expression, inhibited cell growth, induced cell cycle arrest and enhanced cellular senescence. Conversely, overexpression of TRIB2 down-regulated p21 expression, accelerated proliferation and cell cycle, and blocked senescence (Fig. 3 f-j). These findings indicate that TRIB2 regulates proliferation and cellular senescence in a p53-independent manner.

### TRIB2 interacts with AP4 and enhances AP4 transcriptional activities

**Fig. 4:**
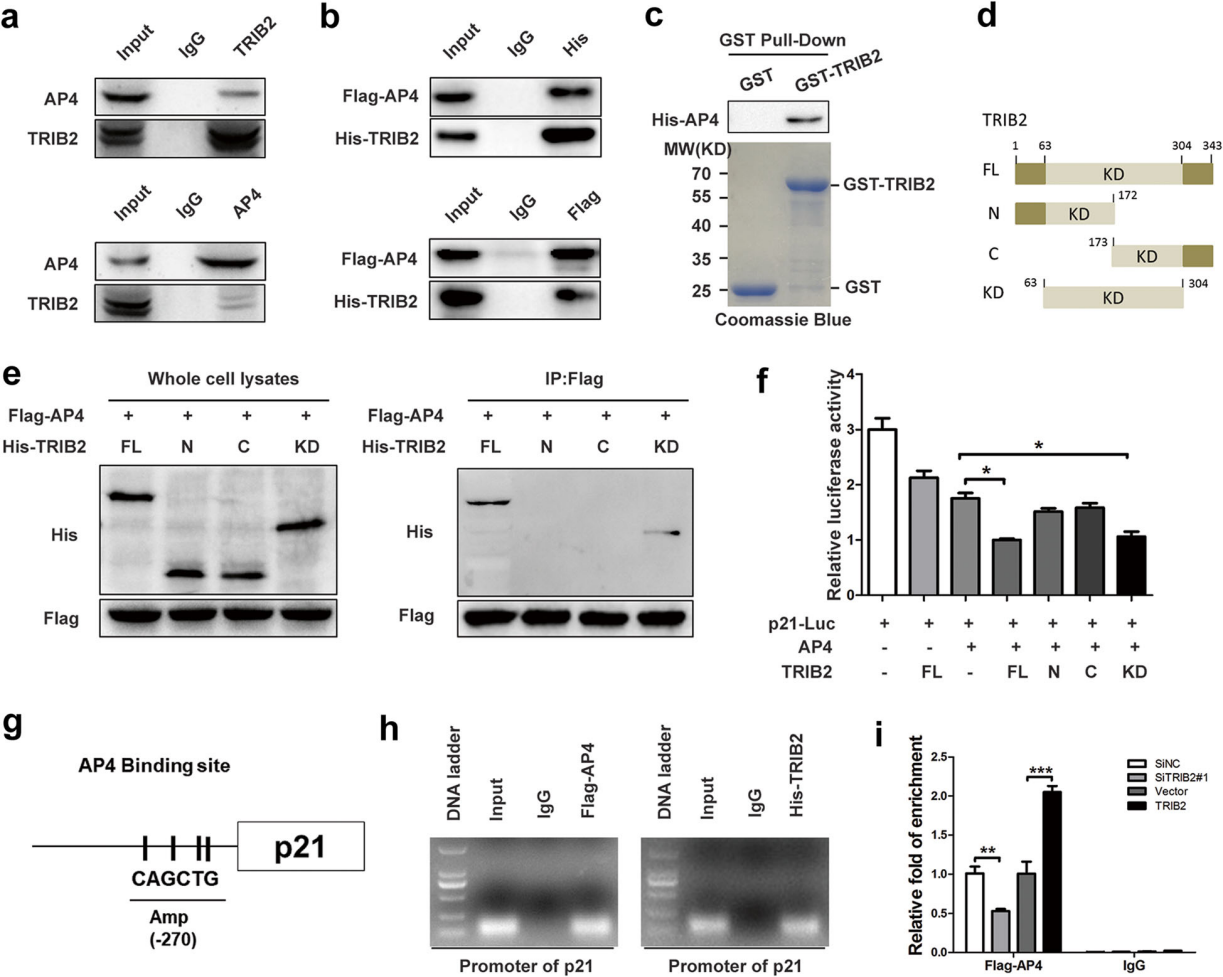
TRIB2 interacts with AP4 and regulates the function of AP4. **a** co--IP analysis with anti-TRIB2 or anti-AP4 antibody in SW48 cells. **b** co-IP analysis with anti-Flag or anti-His antibody in HEK 293 T cells. HEK 293 T cells were transfected with Flag-AP4 and His-TRIB2. After 48 h of transfection, whole cell lysates were immunoprecipitated with an anti-Flag or anti-His antibody and blotted with an anti-His or anti-Flag antibody, respectively. **c** In vitro GST pull down assay to verify the binding of TRIB2 and AP4. Purified His-AP4 protein was incubated with GST or GST-TRIB2 bound to glutathione-Sepharose beads at 4 °C, and the interaction was confirmed by western blot (up panel); the expression of GST and GST-TRIB2 was confirmed by coomassie blue staining (down panel). **d** and **e** Mapping of TRIB2 regions binding to AP4. Flag-AP4 was co-transfected with TRIB2-deleption mutants into HEK293T cells. After 48 h of transfection, the whole cell lysates were immunoprecipitated with anti-Flag antibody and blotted with an anti-His antibody. **f** luciferase activities of p21 promoter in SW48 cells transiently transfected with p21-luc, Flag-AP4 and His-TRIB2 or its truncated mutants, as indicated. g and h ChIP analysis of AP4 and TRIB2 binding at the p21 promoter. SW48 cells were transfected with Flag-AP4 or His-TRIB2. After 48 h of transfection, the chromatin complexes from transfected SW48 cells were subjected to immunoprecipitate with Flag or His antibody overnight for ChIP assay. i ChIP analysis of AP4 binding at the Amp (− 270) region of p21 promoter in TRIB2 knockdown or overexpressed SW48 cells. Results are presented as mean ± SD from three independent assays, * *p* < 0.05, ** *p* < 0.01, *** *p* < 0.001, t-test

The transcription factor AP4 belongs to bHLZ family, and has an important role in cell growth and development. It forms homodimers and binds to the E-box motif CAGCTG to repress viral and cellular genes [25, 26]. The expression of AP4 was elevated in CRC tumor tissues compared with their corresponding normal tissues (9/15; Additional file 4: Figure S4a). AP4 has been shown to induce cell cycle arrest and repress p21 expression via occupying four CAGCTG motifs in the p21 promoter [21]. Deletion of AP4 by AP4-specific siRNA (siAP4#1 and siAP4#2) significantly increased mRNA and protein levels of p21, inhibited cell proliferation and promoted cellular senescence in SW48 and LoVo cells (Additional file 4: Figure S4b, c, d and e). Data from mass spectrometry (BioGRID) identify a number of proteins including TRIB2 as partners of AP4. To confirm the TRIB2/AP4 interaction, we carried out co-IP assay using SW48 cells or HEK 293 T cells co-transfected with Flag-AP4 and His-TRIB2. AP4 and TRIB2 were found to precipitate with each other (Fig. 4 a and b). Besides, pull down assay verified that AP4 band to GST-TRIB2 in vitro but not GST protein alone (Fig. 4 c). Next we constructed a series of different deletion mutants of His-TRIB2, and transfected them with Flag-AP4 into HEK 293 T cells. co-IP results indicated that it was kinase-like domain (KD) of TRIB2 interacted with AP4 (Fig. 4 d and e).

TRIBs are members of the pseudokinase family, and position protein ‘substrates’ and control their E3 ligase-dependent ubiquitination [27]. Thus we hypothesized that TRIB2 regulated p21 expression and senescence predominantly through regulating AP4 expression. However, as shown in Additional file 2: Figure S2a and b, TRIB2 could not directly regulate the expression of AP4. Recent studies have shown that TRIB3 binds to some transcription factor, such as ATF4 and PPARγ, and regulates their transcription activities [28, 29]. So we supposed that TRIB2 might regulate AP4 mediated transcription and performed luciferase assay using p21-Luc. The results showed that overexpression of AP4 suppressed and co-transfection of TRIB2 further suppressed the activities of p21 promoter (Fig. 4f), which indicated TRIB2 enhanced the function of AP4. Further study confirmed that it was kinase-like domain (KD) of TRIB2, but not the N- or C-terminus, that promoted the inhibitory effect of AP4 on p21 promoter activities (Fig. 4f).

To further address the mechanism of how TRIB2 regulated the function of AP4, we conducted ChIP assays on SW48 cells that ectopically expressed Flag-AP4. The results showed that Flag-AP4 was enriched in the proximal region (− 218 to − 324) of the p21 gene promoter (Fig. 4h, left panel). Next, we performed ChIP assays on SW48 cells ectopically expressing His-TRIB2, and found that His-TRIB2 was enriched in the same region with Flag-AP4 (Fig. 4h, right panel). To elucidate how TRIB2 affected the binding of AP4 on p21 promoter, we performed ChIP experiments in TRIB2 knockdown or overexpressed SW48 cells. The results indicated that knockdown of TRIB2 decreased and overexpression of TRIB2 increased AP4-binding on p21 promoter (Fig. 4i). Taken together, TRIB2 physically interacts with AP4 and enhances binding of AP4 on p21 promoter to negatively regulate p21 expression.

### Enhanced transcription activities of AP4 by TRIB2 accelerates CRC cells proliferation and hinders cellular senescence

**Fig. 5:**
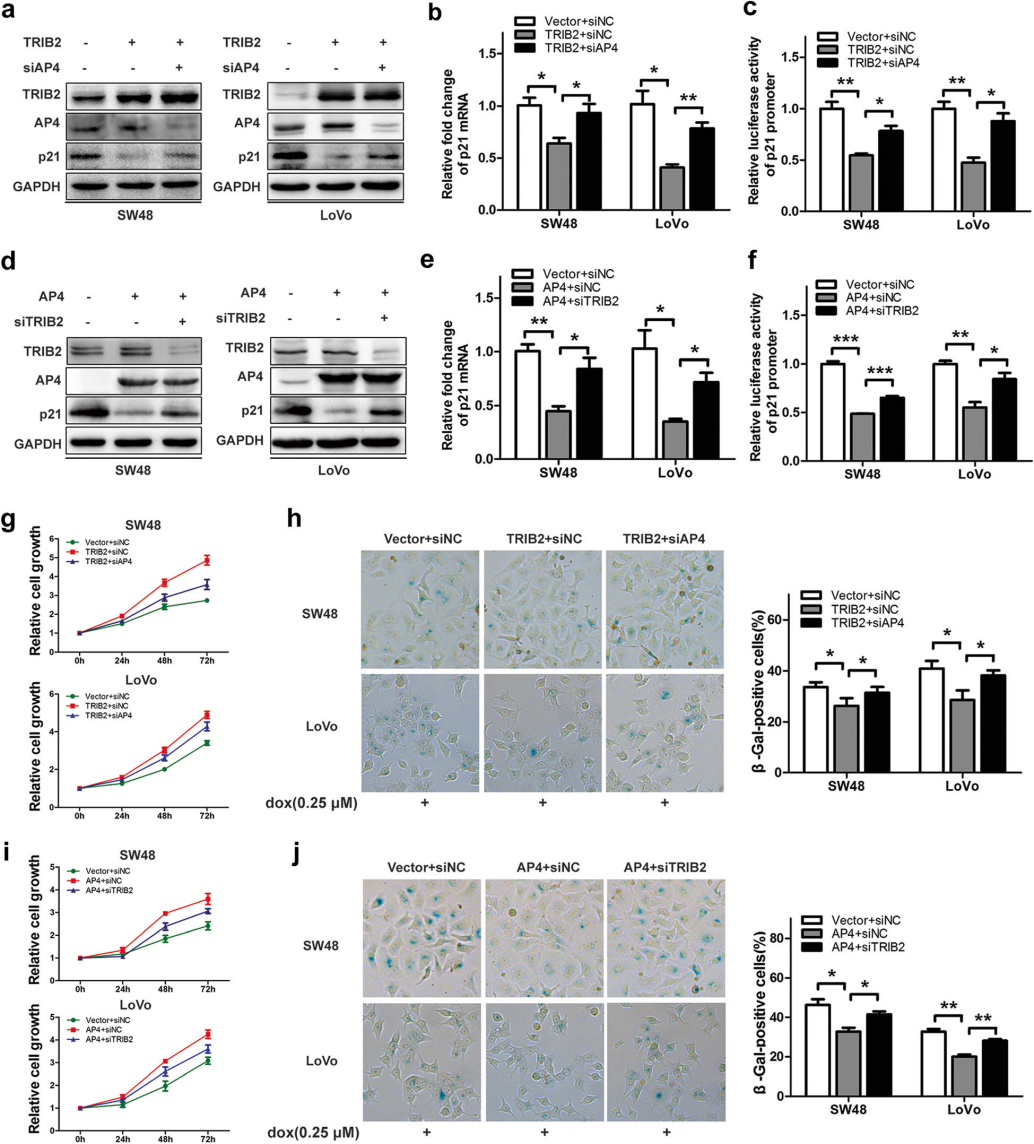
TRIB2 regulates cell proliferation and cellular senescence dependent on AP4. **a** Western blot analysis of TRIB2, AP4 and p21 expression in SW48 and LoVo cells transfected with TRIB2-expressing plasmid and or not AP4-specific siRNA. **b** mRNA expression of p21 in SW48 and LoVo cells transfected with TRIB2-expressing plasmid and or not AP4-specific siRNA. **c** p21-Luc was co-transfected with TRIB2-expressing plasmid and or not AP4-specific siRNA into SW48 and LoVo cells. After 48 h of transfection, the cells were harvest and luciferase activities were measured. **d** Western blot of TRIB2, AP4 and p21 expression in SW48 and LoVo cells transfected with AP4-expressing plasmid and or not TRIB2-specific siRNA. **e** mRNA expression of p21 in SW48 and LoVo cells transfected with AP4-expressing plasmid and or not TRIB2-specific siRNA, respectively. **f** p21-Luc was co-transfected with AP4-expressing plasmid and or not TRIB2-specific siRNA into SW48 and LoVo cells. After 48 h of transfection, the cells were harvest and luciferase activities were measured. **g** Cell proliferation analysis of TRIB2-overexpressed SW48 and LoVo cells transfected with or without AP4-specific siRNA. **h** SA-β-gal staining analysis in TRIB2 overexpressed SW48 and LoVo cells transfected with or without AP4-specific siRNA (dox, 0.25 μmol/l, 48 h). The percentage of SA-β-gal-positive cells was analyzed (right panel). **i** Cell proliferation analysis of AP4-overexpressed SW48 and LoVo cells transfected with or without TRIB2-specific siRNA. **j** SA-β-gal staining analysis in AP4-overexpressed SW48 and LoVo cells transfected with or without TRIB2-specific siRNA (dox, 0.25 μmol/l, 48 h). The percentage of SA-β-gal-positive cells was analyzed (right panel). Results are presented as mean ± SD from three independent assays, * *p* < 0.05, ** *p* < 0.01, *** *p* < 0.001, t-test

In view of results above, we investigated the role of TRIB2/AP4 interaction in tumorigenicity. To demonstrate whether AP4 was critical in TRIB2-induced suppression of p21 expression, we overexpressed TRIB2 with or without AP4 knockdown and detected the protein and mRNA levels of p21. Elevated TRIB2 markedly repressed p21 expression in SW48 and LoVo cells, and this could be reversed by AP4 knockdown (Fig. 5a and b). The luciferase assay indicated TRIB2 inhibited p21 promoter activities in an AP4-dependent manner (Fig. 5c). We then performed CCK8 and SA-β-gal staining analysis to examine the effects of AP4 on TRIB2-mediated functions, and found that silencing AP4 could significantly block TRIB2 overexpression-promoted SW48 and LoVo cells growth and enhance TRIB2 overexpression-inhibited cellular senescence (Fig. 5g and h).

Next, we investigated the importance of TRIB2 in modulating AP4 functions, and co-transfected AP4 expressing plasmid with or without TRIB2-specific siRNA in SW48 and LoVo cells. As shown in Fig. 5d, the decrease of p21 protein levels caused by elevated AP4 was abrogated by TRIB2 knockdown. The RT-PCR and luciferase assay indicated that the transcription activities of AP4 were dependent on TRIB2/AP4 interaction (Fig. 5e and f). We then examined the role of TRIB2/AP4 interaction in AP4-mediated tumorigenesis. Data from CCK8 and SA-β-gal staining analysis showed that the functions of AP4 in cell proliferation were markedly impaired and repressed cellular senescence by AP4 was enhanced in the absence of TRIB2 (Fig. 5i and j). Taken together, these results reveal that TRIB2 promotes cancer cells growth and suppresses cellular senescence via enhancing AP4 transcription activities.

### Silencing of TRIB2 enhances cellular senescence and inhibits cell proliferation in vivo

**Fig. 6:**
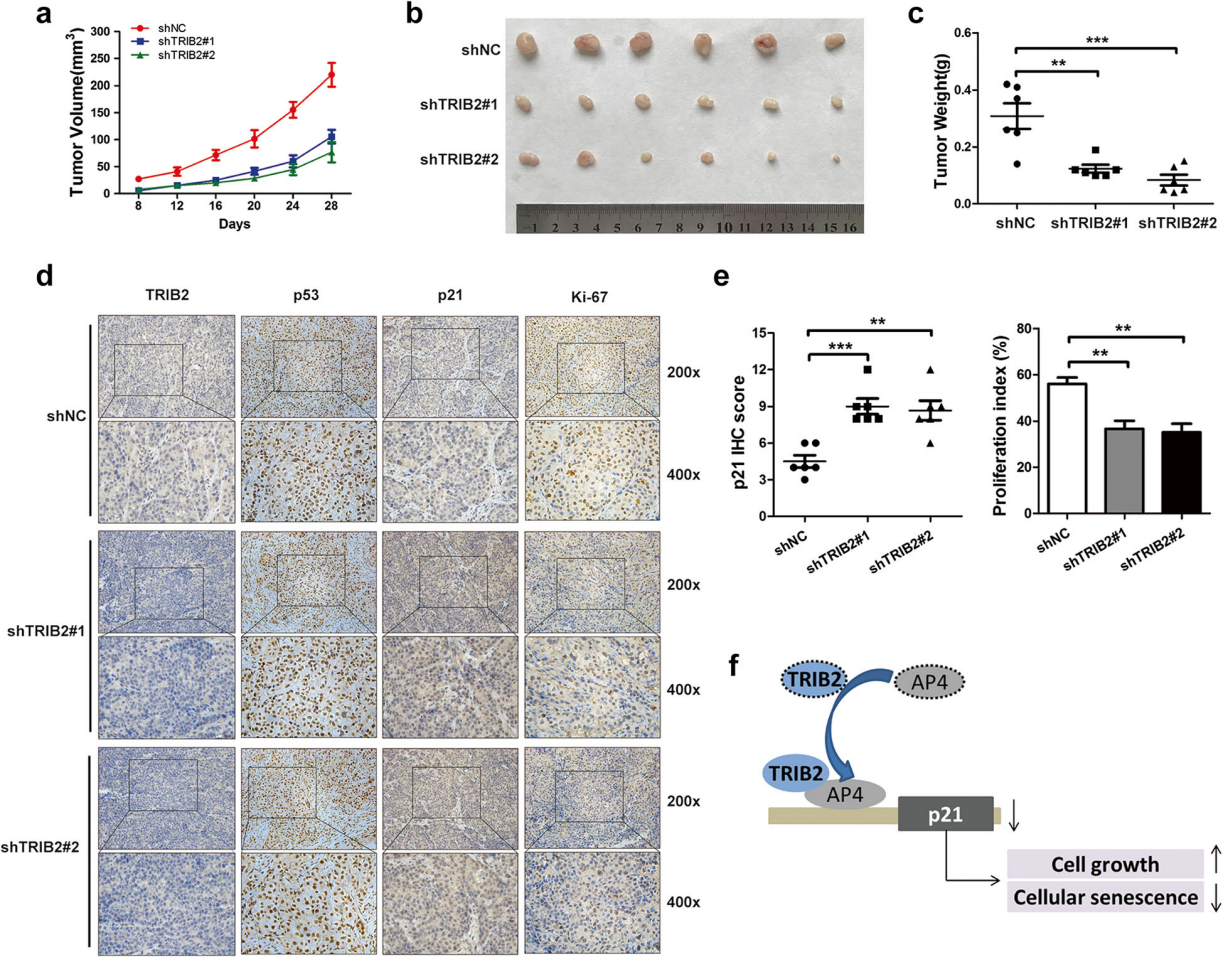
TRIB2 knockdown inhibits cell proliferation and enhances cellular senescence in vivo. **a** Tumor volumes of the TRIB2-knockdown and control groups were calculated every 4 days. **b** and **c** Tumor size and weight of the TRIB2-knockdown and control groups. **d** IHC staining of TRIB2, p53, p21 and Ki-67 in the indicated xenograft tumors. **e** Quantification of p21 expression, and proliferation index in the indicated xenograft tumors, proliferation index was determined using the percentage of Ki-67 positive cells. The results were presented as mean ± SD, *n* = 6, * *p* < 0.05, ** *p* < 0.01, *** *p* < 0.001, t-test. **f** Proposed model: TRIB2/AP4 interaction enhances binding of AP4 on p21 promoter to negatively regulate p21 expression, which consequently leads to increased cell growth and inhibited cellular senescence in CRC

To evaluate the role of TRIB2 in regulation of senescence and cell proliferation in vivo, we established the CRC xenograft mice model. The stable TRIB2-knockdown SW48 cells (shTRIB2#1 and shTRIB2#2) or shNC cells were subcutaneously injected into nude mice (1 million cells per mouse). When the tumors became visible to naked eyes after about a week, we calculated tumors volume every 4 days and plotted a tumor growth curve. As shown in Fig. 6a, tumor volumes of the shTRIB2 groups grew slower compared with control group. All mice were killed 28 days after cancer cells injection. We then measured the tumor weights and gained a similar conclusion (Fig. 6b and c). IHC analysis showed that the number of Ki-67 positive cells was dramatically decreased in shTRIB2 groups. Consistent with the above results, p21 expression level was significantly elevated in tumors formed by stable TRIB2-knockdown cells (Fig. 6e). However, no difference on p53 expression was found between the two groups (Fig. 6d). The results support the notion that TRIB2 plays a vital role in cancer cell proliferation and cellular senescence in a p53-independent manner.

To further confirm the role of AP4 in TRIB2-mediated tumorigenesis in vivo, we established xenografts by subcutaneous injection of SW48 cells overexpressing TRIB2 with or without AP4 knockdown. Tumors originated from TRIB2 overexpressed cells grew significantly faster than vector cells, and this effect was impaired by silencing AP4 (Additional file 5: Figure S5a). Consistent with the result, tumor size and weight of TRIB2 overexpressed group were larger than that of the vector group and TRIB2 plus shAP4 group (Additional file 5: Figure S5b and c). IHC staining showed similar results (Additional file 5: Figure S5d and e). Taken together, these data suggest that the effects of TRIB2 to the cell proliferation and cellular senescence are dependent on AP4 in vivo.

## Discussion

Tribbles, members of the pseudokinase family, were demonstrated to regulate many cellular functions, including glucose and lipid metabolism, apoptosis, adipocyte differentiation and inflammation. Despite possessing no kinase activity, tribbles act as molecule adaptors to regulated protein-protein interaction [2]. Recently, growing evidence suggests that tribbles act as pro-oncogenes and play critical roles in some kinds of malignant tumors [7, 30, 31]. For instance, elevated TRIB1 expression is able to induce AML via activation of ERK and degradation of C/EBPα; TRIB3 regulates MAPK- and TGF-β-mediated Notch activation to promote cancer progression in breast cancer cells and promotes APL progression via interacting with and inhibiting degradation of oncoprotein PML-RARα [32, 33]; TRIB2 is also reported to contribute to acute myeloid leukemia (AML) and hematopoietic development [34]. In the present study, we demonstrated that TRIB2 expression in human CRC tissues was much higher than that in adjacent tissues and negatively correlated with prognosis of CRC patients, which is consistent with the results reported by Hill, R. et al. [10]. Moreover, we identified TRIB2 as an independent prognostic factor affecting the survival of CRC patients through multivariate analysis, further illustrating its important clinical role in CRC patients. These results indicated that TRIB2 might be an available prognostic marker for CRC patients.

Studies on the mechanism of regulating the expression of TRIB2 have been widely conducted, and transcriptional regulation is likely to be pivotal in TRIB2 expression. The aberrant expression of some oncogenes has been reported to account for elevated TRIB2. Wang et al. showed that the TRIB2 is a direct target of Wnt/TCF pathway in liver cancer [8]. In AML cells, dysregulated C/EBPalpha and E2F1 contributes to up-regulation of TRIB2 [35]. In human T cell acute lymphoblastic leukemia (T-ALL), NOTCH, PITX and TAL1 are also found to up-regulate TRIB2 [36]. However, it is unclear why TRIB2 is overexpressed in CRC patients. It would be interesting to further investigate the mechanism of TRIB2 up-regulation in CRC.

To further explore the oncogenic role of TRIB2 in tumorigenesis, we performed a series of experiments in CRC cell lines and found that ectopic expression of TRIB2 dramatically blocked cellular senescence. Cellular senescence is identified as a state of irreversible cell-cycle arrest induced by a series of insults, and induction of senescence is considered as a potent strategy for suppressing cancer progression with minor side effects [37]. As the critical marker for cellular senescence, p21 has been proved to drive senescence initiation. Various stimuli including ROS, mitochondrial dysfunction, irradiation and chemotherapeutic drugs induce cellular senescence through DNA damage response by activating ATM and eventually activating p53/p21 pathway [38]. Although p21 is mainly regulated by p53 at the transcription level, multiple transcription factors (BRCA1, Smad3, AP4 and c-myc) have also been reported to control the transcriptional activation or repression of p21 [39–41].In our study, TRIB2 could negatively regulate p21 promoter activities and expression, and inhibit cellular senescence in CRC cells. Earlier reports of Richard et al. have showed that in melanoma cells TRIB2 inhibits p53 and p21 expression in an AKT-dependent manner [4]. However, in the current study we did not detect significant changes of p53 expression in SW48 cells when TRIB2 was silenced. Experiments in CRC cells, which were transfected with p53- and or not TRIB2-specific siRNA, suggested that TRIB2 knockdown could still transcriptionally increase p21 expression even in the absence of p53. These results revealed that TRIB2 regulated p21 expression in a p53-independent manner, which is opposite with previous study. We speculated that TRIB2 may have distinct functions depending on etiologic background of different cancer types.

To extensively study the mechanisms responsible for TRIB2-mediated regulation of cellular senescence, we analyzed the BioGRID database and found TRIB2 directly interacted with transcription factor AP4. AP4, belonging to the basic helix-loop-helix transcription factors (bHLH-LZ) superfamily, is identified as an oncogene and accelerates development and progression in a variety of human cancers [23, 42–45]. Moreover, AP4 is frequently up-regulated in these malignancies and positively correlates with poor prognosis. The functions of AP4 in modulating tumorigenic properties have been extensively studied. It plays a critical role in regulating stem-like phenotypes and epithelial-mesenchymal transition (EMT) through directly targeting genes such as CD44, LGR5, VIM and E-cadherin [45]. Mechanically, AP4 recognizes and binds to consensus E-box motif CAGCTG in promoters of downstream target genes [22]. There is evidence that AP4 directly binds to p21 promoter and transcriptionally represses p21 expression [21]. Our results suggest that TRIB2 regulates p21 expression by enhancing the functions of AP4, through protein-protein interaction. Similar to our results, Nobumichi et al. showed that TRIB3, another member of the tribbles family, interacts with transcription factor ATF4 and then regulates transcriptional activity of ATF4 [29]. In addition, TRIB3 alters PPARγ transcriptional activity through directly binding to PPARγ during adipocyte differentiation [28]. The above evidence indicates that it is a common phenomenon for the tribble family members to influence the activity of transcription factors through protein-protein interaction. However, the exact mechanism of how TRIB2 regulate the transcription activities of AP4 remains to be clarified. It is likely that TRIB2 acts as scaffold to recruit some corepressor (s) or trigger histone modification. Future studies are needed to determine the specific mechanism.

## Conclusions

In summary, we noted the oncogenic effect of TRIB2 in human colorectal cancer. The increased level of TRIB2 promotes proliferation and induces cell cycle arrest by inhibiting cellular senescence, and this is mediated by TRIB2/AP4 interaction which leads to enhanced AP4 transcription activity. The present study expands our understanding of TRIB2 in cellular senescence, and suggests that TRIB2/AP4/p21 pathway is a potential target for tumor therapeutic intervention.

## Additional files

Additional file 1: **Figure S1.** The functions of TRIB2 on cell growth are not due to cell apoptosis in CRC. a and b Flow cytometry analysis of the percentage of apoptotic cells for SW48 and LoVo cells transfected with siTRIB2 (#1 and #2) or siNC for 72 h. (TIF 1224 kb)

Additional file 2: **Figure S2.** TRIB2 could not affect cyclin D1 and p16 expression.a Western blot analysis of Cyclin D1 and p16 expression in SW48 and LoVo cells transfected with siTRIB2 (#1 and #2) or siNC. b Western blot analysis of Cyclin D1 and p16 expression in SW48 and LoVo cells transfected with TRIB2 expressing plasmid or vector. (TIF 1118 kb)

Additional file 3: **Figure S3.** Overexpression of TRIB2 in CRC cells promotes tumor cell growth and inhibits cellular senescence. a Western blot analysis of TRIB2 in SW48 and LoVo cells transfected with TRIB2-expressing plasmid or vector. b Cell viability of TRIB2-overexpressed or control SW48 and LoVo cells at 0, 24, 48, 72 h, respectively. c Cell cycle distribution by flow cytometry detection in TRIB2-overexpressed or control SW48 and LoVo cells; d SA-β-gal staining analysis of TRIB2-overexpressed or control SW48 and LoVo cells treated with dox (0.25 μmol/l, 48 h, left panel, representative images of SA-β-gal staining). e Western blot analysis of TRIB2, p53 and p21 in SW48 and LoVo cells transfected with TRIB2-expressing plasmid or vector. f RT-PCR analysis of p53 and p21 expression in SW48 and LoVo cells transfected with TRIB2-expressing plasmid or vector. g Relative luciferase activity of p21 in SW48 and LoVo cells transiently transfected with p21-Luc plus TRIB2-expressing plasmid or vector. Results are presented as mean ± SD from three independent assays, * *p* < 0.05, ** *p* < 0.01, *** *p* < 0.001, t-test. (TIF 2282 kb)

Additional file 4: **Figure S4.** AP4 promotes tumorigenesis in colorectal cancer. a mRNA expression of AP4 in 15 pairs of human primary CRC tissues (normal and Tumor). b Western blot analysis of AP4 and p21 in SW48 and LoVo cells transfected with AP4 specific siRNA (#1 and #2) or siNC. c RT-PCR analysis of AP4 and p21 in SW48 and LoVo cells transfected with AP4 specific siRNA (#1 and #2) or siNC. d CCK8 analysis of cell proliferation capacity in AP4-knockdown or control SW48 and LoVo cells. e SA-β-gal staining analysis of SW48 and LoVo cells transfected with AP4 specific siRNA (#1 and #2) or siNC (dox, 0.25 μmol/l, 48 h). left panel, representative images of SA-β-gal staining; right panel, percentage of SA-β-gal-positive cells. Results are presented as mean ± SD from three independent assays, * *p* < 0.05, ** *p* < 0.01, *** *p* < 0.001, t-test. (TIF 2707 kb)

Additional file 5: **Figure S5** The functions that TRIB2 mediated in vivo are dependent on AP4. a Tumor volumes of TRIB2-overexpressed or TRIB2-overexpressed plus AP4 knockdown or control groups were calculated every 4 days. b and c Tumor size and weight of tumors generated from TRIB2-overexpressed SW48 cells transfected with or without AP4-specific siRNA and control cells. d IHC staining of TRIB2, p53, p21 and Ki-67 in the indicated xenograft tumors. e Quantification of p21 expression, and proliferation index in the indicated xenograft tumors, proliferation index was determined using the percentage of Ki-67 positive cells. The results were presented as mean ± SD, *n* = 6, * *p* < 0.05, ** *p* < 0.01, *** *p* < 0.001, t-test. (TIF 4363 kb)

## Abbreviations

AML: Acute myeloid leukemia
AP4 CDK: Cyclin-dependent kinases
AP4: Transcription factor
CDKN1A: Cyclin dependent kinase inhibitor 1A
ChIP: Chromatin immunoprecipitation
co-IP: Co-immunoprecipitation
CRC: Colorectal cancer
DDR: DNA damage response
FBS: Fetal bovine serum
GEO: Gene Expression Omnibus
IHC: Immunohistochemistry
RNA TCGA: The Cancer Genome Atlas
SA-β-gal: Senescence-associated β-galactosidase
siRNA: Short interfering
TRIB2: Tribbles pseudokinase 2
