# Supplementary figures and images for: TRIB2 functions as novel oncogene in colorectal cancer by blocking cellular senescence through AP4/p21 signaling
Source: Mol Cancer. 2018 Dec 12;17:172. doi: 10.1186/s12943-018-0922-x (PMC6291992; doi:10.1186/s12943-018-0922-x)

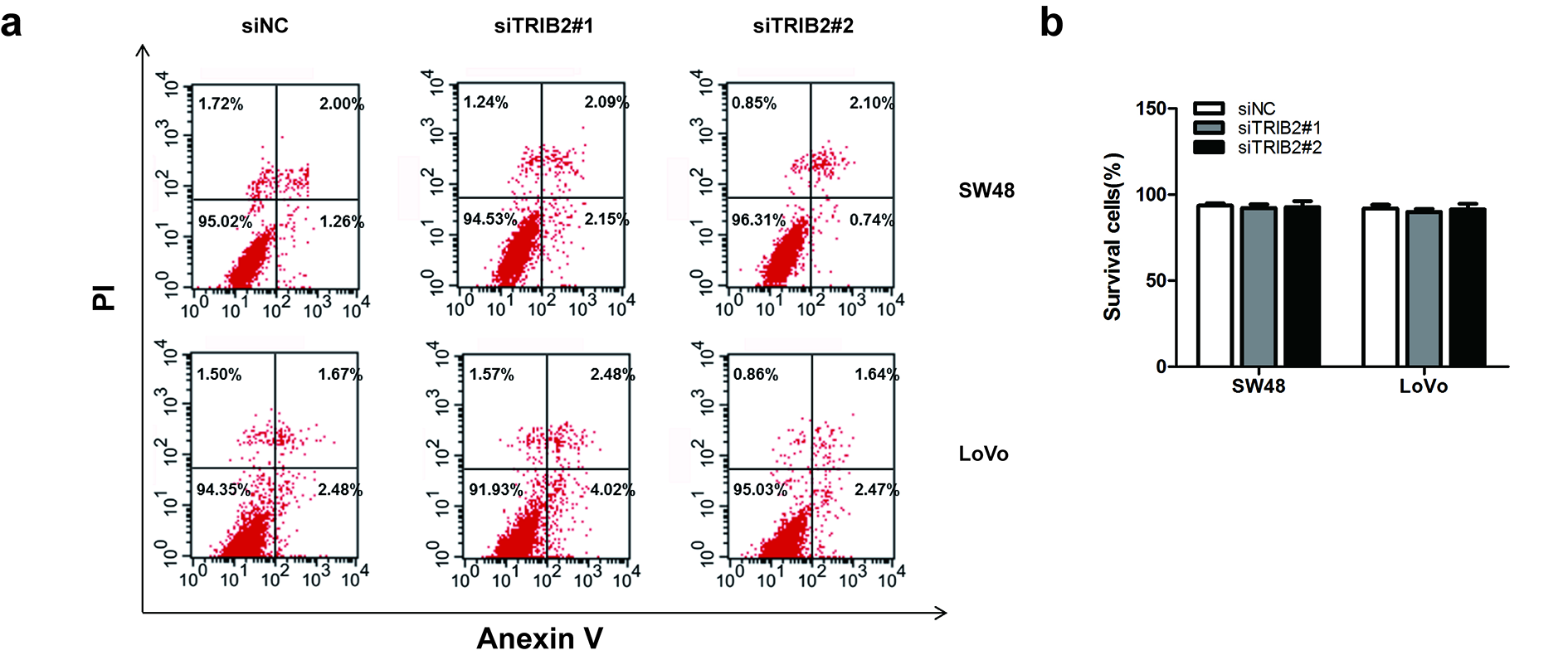

Supplement: Supplementary file 1 — Figure S1. The functions of TRIB2 on cell growth are not due to cell apoptosis in CRC. a and b Flow cytometry analysis of the percentage of apoptotic cells for SW48 and LoVo cells transfected with siTRIB2 (#1 and #2) or siNC for 72 h. (TIF 1224 kb) [file 12943_2018_922_MOESM1_ESM.tif]

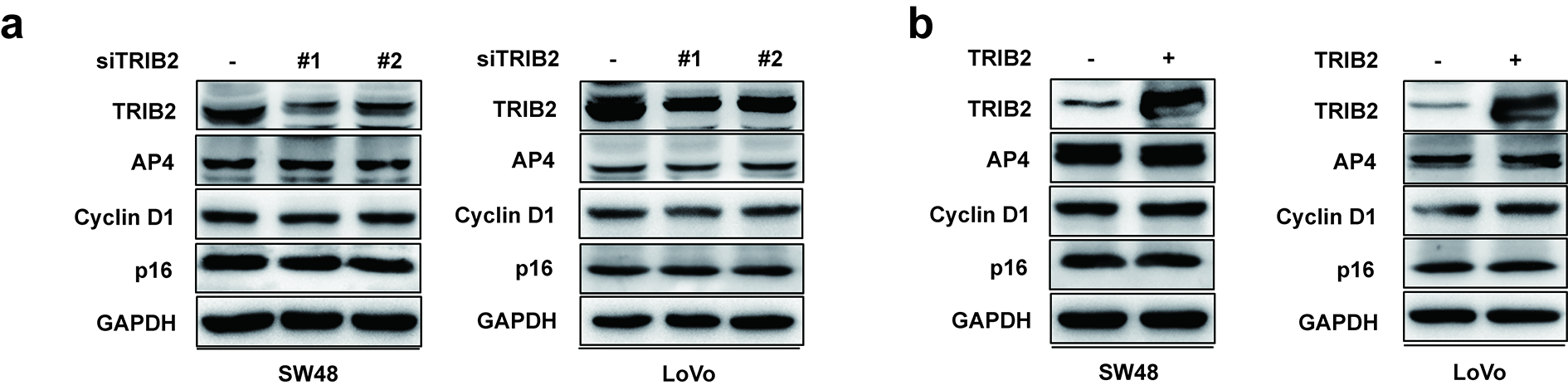

Supplement: Supplementary file 2 — Figure S2. TRIB2 could not affect cyclin D1 and p16 expression.a Western blot analysis of Cyclin D1 and p16 expression in SW48 and LoVo cells transfected with siTRIB2 (#1 and #2) or siNC. b Western blot analysis of Cyclin D1 and p16 expression in SW48 and LoVo cells transfected with TRIB2 expressing plasmid or vector. (TIF 1118 kb) [file 12943_2018_922_MOESM2_ESM.tif]

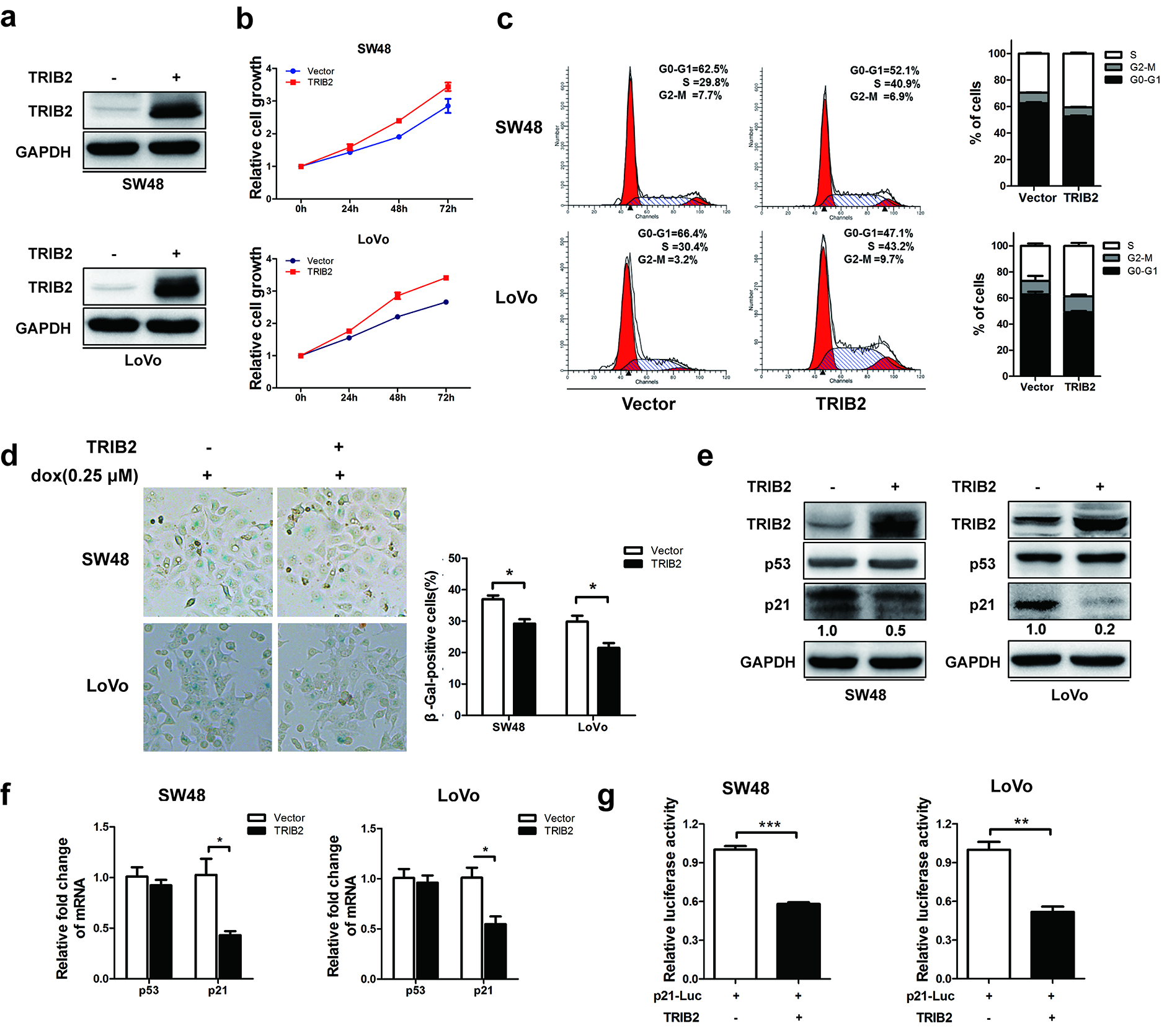

Supplement: Supplementary file 3 — Figure S3. Overexpression of TRIB2 in CRC cells promotes tumor cell growth and inhibits cellular senescence. a Western blot analysis of TRIB2 in SW48 and LoVo cells transfected with TRIB2-expressing plasmid or vector. b Cell viability of TRIB2-overexpressed or control SW48 and LoVo cells at 0, 24, 48, 72 h, respectively. c Cell cycle distribution by flow cytometry detection in TRIB2-overexpressed or control SW48 and LoVo cells; d SA-β-gal staining analysis of TRIB2-overexpressed or control SW48 and LoVo cells treated with dox (0.25 μmol/l, 48 h, left panel, representative images of SA-β-gal staining). e Western blot analysis of TRIB2, p53 and p21 in SW48 and LoVo cells transfected with TRIB2-expressing plasmid or vector. f RT-PCR analysis of p53 and p21 expression in SW48 and LoVo cells transfected with TRIB2-expressing plasmid or vector. g Relative luciferase activity of p21 in SW48 and LoVo cells transiently transfected with p21-Luc plus TRIB2-expressing plasmid or vector. Results are presented as mean ± SD from three independent assays, * p < 0.05, ** p < 0.01, *** p < 0.001, t-test. (TIF 2282 kb) [file 12943_2018_922_MOESM3_ESM.tif]

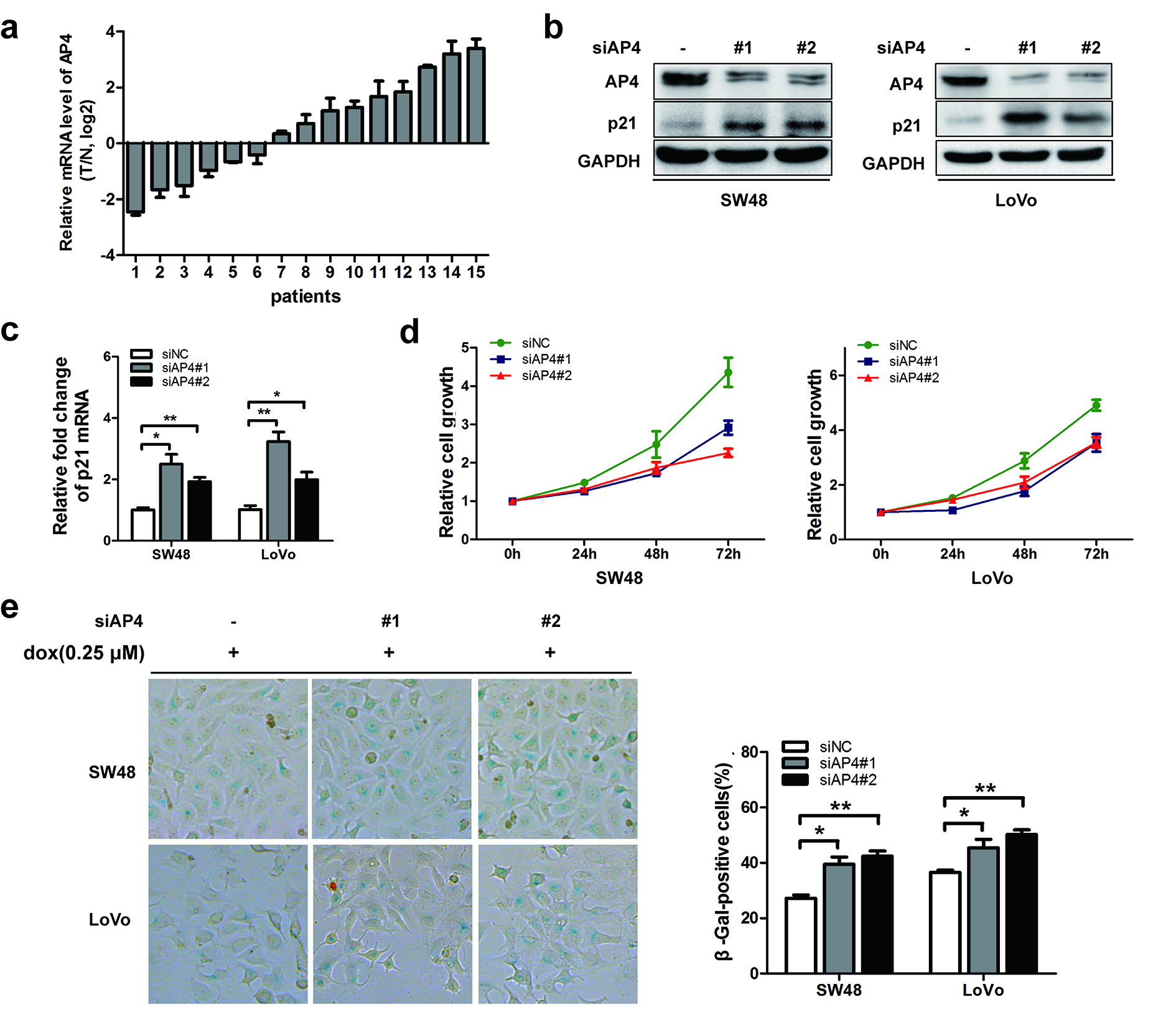

Supplement: Supplementary file 4 — Figure S4. AP4 promotes tumorigenesis in colorectal cancer. a mRNA expression of AP4 in 15 pairs of human primary CRC tissues (normal and Tumor). b Western blot analysis of AP4 and p21 in SW48 and LoVo cells transfected with AP4 specific siRNA (#1 and #2) or siNC. c RT-PCR analysis of AP4 and p21 in SW48 and LoVo cells transfected with AP4 specific siRNA (#1 and #2) or siNC. d CCK8 analysis of cell proliferation capacity in AP4-knockdown or control SW48 and LoVo cells. e SA-β-gal staining analysis of SW48 and LoVo cells transfected with AP4 specific siRNA (#1 and #2) or siNC (dox, 0.25 μmol/l, 48 h). left panel, representative images of SA-β-gal staining; right panel, percentage of SA-β-gal-positive cells. Results are presented as mean ± SD from three independent assays, * p < 0.05, ** p < 0.01, *** p < 0.001, t-test. (TIF 2707 kb) [file 12943_2018_922_MOESM4_ESM.tif]

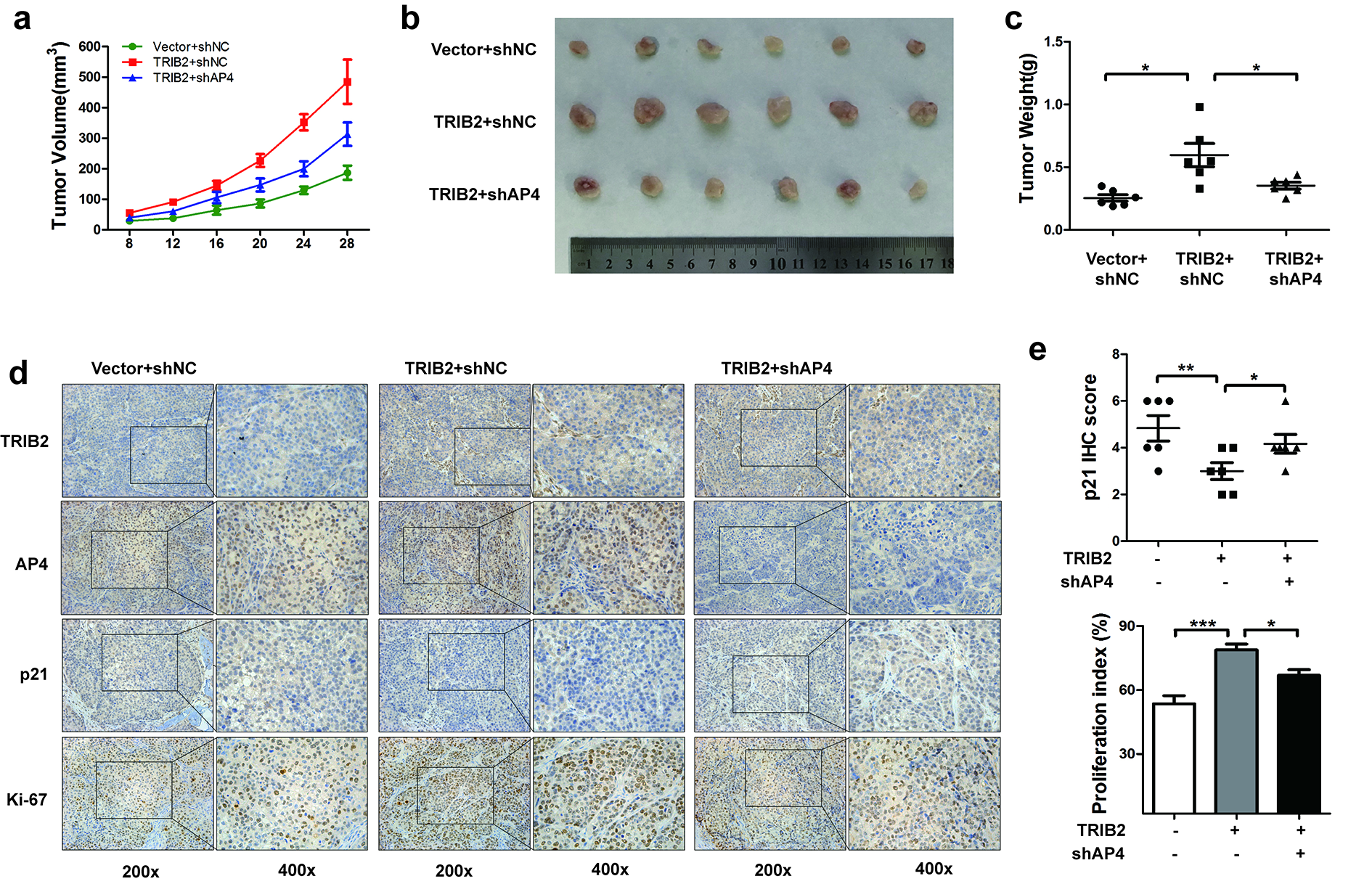

Supplement: Supplementary file 5 — Figure S5 The functions that TRIB2 mediated in vivo are dependent on AP4. a Tumor volumes of TRIB2-overexpressed or TRIB2-overexpressed plus AP4 knockdown or control groups were calculated every 4 days. b and c Tumor size and weight of tumors generated from TRIB2-overexpressed SW48 cells transfected with or without AP4-specific siRNA and control cells. d IHC staining of TRIB2, p53, p21 and Ki-67 in the indicated xenograft tumors. e Quantification of p21 expression, and proliferation index in the indicated xenograft tumors, proliferation index was determined using the percentage of Ki-67 positive cells. The results were presented as mean ± SD, n = 6, * p < 0.05, ** p < 0.01, *** p < 0.001, t-test. (TIF 4363 kb) [file 12943_2018_922_MOESM5_ESM.tif]
